# EMG Processing Based Measures of Fatigue Assessment during Manual Lifting

**DOI:** 10.1155/2017/3937254

**Published:** 2017-02-19

**Authors:** E. F. Shair, S. A. Ahmad, M. H. Marhaban, S. B. Mohd Tamrin, A. R. Abdullah

**Affiliations:** ^1^Department of Electrical and Electronic Engineering, Faculty of Engineering, Universiti Putra Malaysia (UPM), Selangor, Malaysia; ^2^Faculty of Electrical Engineering, Universiti Teknikal Malaysia Melaka (UTeM), Melaka, Malaysia; ^3^Department of Environmental and Occupational Health, Faculty of Medicine and Health Sciences, Universiti Putra Malaysia (UPM), Selangor, Malaysia

## Abstract

Manual lifting is one of the common practices used in the industries to transport or move objects to a desired place. Nowadays, even though mechanized equipment is widely available, manual lifting is still considered as an essential way to perform material handling task. Improper lifting strategies may contribute to musculoskeletal disorders (MSDs), where overexertion contributes as the highest factor. To overcome this problem, electromyography (EMG) signal is used to monitor the workers' muscle condition and to find maximum lifting load, lifting height and number of repetitions that the workers are able to handle before experiencing fatigue to avoid overexertion. Past researchers have introduced several EMG processing techniques and different EMG features that represent fatigue indices in time, frequency, and time-frequency domain. The impact of EMG processing based measures in fatigue assessment during manual lifting are reviewed in this paper. It is believed that this paper will greatly benefit researchers who need a bird's eye view of the biosignal processing which are currently available, thus determining the best possible techniques for lifting applications.

## 1. Introduction

Musculoskeletal disorders (MSDs) caused by manual lifting tasks have been perceived for quite some time as one of the primary work-related injuries which influences the personal satisfaction of industrial workers around the world [1, 2]. Common reasons of MSDs in manual lifting are due to improper lifting and muscle fatigue. National Institute of Occupational Safety and Health (NIOSH) has published a technical report entitled Work Practices Guide for Manual Lifting as a reference to perform correct lifting [3]. However, in terms of muscle fatigue monitoring, researchers are still in search of finding the best processing technique to assess the human muscle condition.

## 2. Manual Lifting

Despite the wide utilization of robots in modern industries, there are still numerous assignments in the industry that are performed physically by humans, such as carrying, lifting, pushing, or pulling. These physical movements of the body often cause MSDs that can be subdivided into more particular and perceived regions, such as back disorders (BDs), upper limb disorders (ULDs), and lower limb disorders (LLDs) [4]. ULDs cover distinctive work-related musculoskeletal dissensions in the arm, wrist, hand, elbow, neck, and shoulder, while LLDs are associated with the lower areas of the body such as feet, legs, and hips. The anatomical zones of the body mostly influenced by these MSDs issues due to manual handling are in the spine and lumbar (back area), and the arm, and wrist (upper limb area). These are further supported by various researches done in previous years on muscle areas investigated during manual lifting and are summarized in Table 1.

Manual errands that are performed improperly by workers in the industries may most of the time bring pain in the lumbar spinae [5]. This situation happens when one individual performs forward flexion; the weight of abdominal area and the load affects the lumbar spinae, which is adjusted by trunk muscles (predominately by erector spinae muscles) [6]. This movement causes the erector spinae muscles to make incredible expansive strengths for adjusting the body. Even though there are numerous assistive gadgets such as lift trucks, trolleys, and derricks that can help reduce loads on the lumbar spinae, however in many cases, these instruments are costly and inconvenient.

Lifting strategies are an important measure during lifting and because physically correct lifting is significant, their impact on musculoskeletal well-being is broadly talked about [22]. The impact of lifting techniques including position width (characterized as the separation between the feet in the average sidelong heading with the sagittal symmetry of position) has been considered, recommending the utilization of a wide position to lessening the burden on the spine [23]. Lifting procedures (stoop or squat and semisquat) can also have a significant effect on spine stacking and steadiness during lifting. Past studies have researched lifting methods in biomechanical terms to give some mediation methodologies and recognize the correct lifting procedures [24, 25]. Squat lifting seems to have less lumbar shear stretch and place less weight on noncontractile connective tissues. Stoop lifting has all the earmarks of being more regular and less exhausting. Semisquat lifting might be a decent trade-off lift, despite the fact that early confirmation recommends perhaps higher lumbar minutes [22].

## 3. Muscle Fatigue Assessment

Muscle fatigue is characterized as the long lasting deterioration of the performance of the human operator to create force [24, 25]. A person's muscle capabilities may differ from one another, but the characteristics should have some resemblance. Past researchers have outlined several features of muscle fatigue such as an increment in amplitude and the transition from high frequency range to low frequency [26].

To assess this muscle fatigue characteristics, various techniques are available, depending on the usage. At this moment, even though the measurement of muscle fatigue by use of invasive means such as blood test (blood lactate level and blood oxygen level) and muscle biopsies (pH of muscle) offers higher accuracy compared to noninvasive techniques, it is unsuitable for some applications such as sports, ergonomics, and occupational therapy [27]. The use of noninvasive approach to acquire muscle signals such as electromyography (EMG), sonomyography (SMG), mechanomyography (MMG), and near infrared spectroscopy (NIRS) for research purposes is considered to be more convenient and easy to use [28]. Every technique has its own advantages and disadvantages based on its applications. For the purpose of assessing muscle physiology during manual lifting, EMG is the most commonly used tool and has been well reported by many researchers [29].

## 4. Electromyography Signal

EMG is the measure of electrical potential present on the skin in consequence to a muscle contraction that represents the neuromuscular activities [26]. It can be measured by two methods: (1) applying electrodes to the skin surface (noninvasive) or (2) intramuscular (invasive) within the muscle. Even though intramuscular EMG (imEMG) provides additional benefits to overcome the drawbacks of surface EMG (sEMG) such as maintaining robust electrode contact with the skin and the capacity to record from profound muscles with little EMG crosstalk [27], sEMG and imEMG have been proven to have equal classification performance for data of comparative nature (unmodulated). However the performance of imEMG diminished, compared to surface, when tested on modulated data [28, 29].

The greater selectivity of imEMG with respect to sEMG is due to wires exposed only at the tip but then again may be a hindrance, since the signal may provide local, rather than global information where imEMG recordings depend on the recruitment of motor units; it may be the case that insufficient information was captured at low amplitude/frequency [27].

## 5. Electromyography Preprocessing

When dealing with EMG signal, noise removal is a very important factor to be taken into consideration. This is due to the sensitive nature of EMG signal itself that different types of noises or artifacts unavoidably contaminate it. These noises are caused by various sources originates from either the skin-electrode interface, hardware that intensifies the signal, or other external sources. Therefore, before proceeding with the analysis and classification of EMG signal, a preprocessing is usually introduced to remove these noises. Some of the noises that may affect the signals are inherent noise in electronics equipment, ambient noise, motion artifact, ECG artifacts, inherent instability of signal, and crosstalk. However, this review only covers three types of noises: motion artifact, ECG noise, and device noise.

### 5.1. Motion Artifact

Cutting edge technology is considerably invulnerable to some of the EMG noises, but not to the motion artifact as it has frequency spectra that are affecting the low frequency part of EMG signal. This motion artifact originates at the electrode-skin interface, where there exist muscle movements underneath the skin, and force impulse that goes through the muscle and the skin underlying the electrodes causing movements [30]. These movements will result in a time-varying voltage across the two electrodes.

To remove the low frequency noise caused by the motion artifact, a study has been conducted to ascertain a reasonable value for the high-pass corner frequency filter. The result of the study recommends the use of a Butterworth filter with a 20 Hz corner frequency and a 12 dB/oct slope [31].

### 5.2. ECG Noise

When EMG signal is measure close to the heart, ECG bursts may pollute the EMG recording, which can affect the analysis and result in misinterpretations [32]. This is a natural relic that is often unavoidable. It can be diminished by proper skin preparation and adjusted position of the ground electrode. To date, other than the conventional ECG removal procedures such as the use of high past filtering, several ECG noise removal techniques have been introduced that can clean these ECG bursts but still maintaining the regular EMG characteristics.

In 2009, Lu et al. came out with a study to evaluate the performance of recursive-least-square adaptive filter in the removal of ECG interference from EMG signal [33]. The result of the study shows that due to fast convergence of the recursive-least-square algorithm, the filter is reliable to effectively remove ECG noise.

Later in 2012, independent component analysis (ICA) was proposed to remove the ECG contamination. The filtering effects are measured in terms of root mean square relative errors and linear envelopes correlation of uncontaminated and contaminated EMG. This technique excellently produces good results when EMG and ECG are statistically independent [34].

### 5.3. Device Noise

Another type of noise that usually affects EMG signal is the noise generated inside the EMG device itself that is commonly referred to as inherent noise in electronics equipment [35]. This noise cannot be completely eliminated as all electronic equipment generates noise [36]. However, the severity of the noise can be reduced using high-quality electronic components.

## 6. Electromyography Processing

Biosignal processing is a critical part in biomedical engineering in order to classify the frequency content of a signal. These signals include EMG, electrocardiography (ECG), and electroencephalography (EEG). Most of these biosignals are nonstationary signals due to its time-varying characteristics [37–39]. The problem of this nonstationary signal is the process of assuming it to be stationary over short-time intervals, where stationary analysis techniques are used. However, this assumption is not always suitable, and further methods for nonstationary processes are needed.

**Figure 1:**
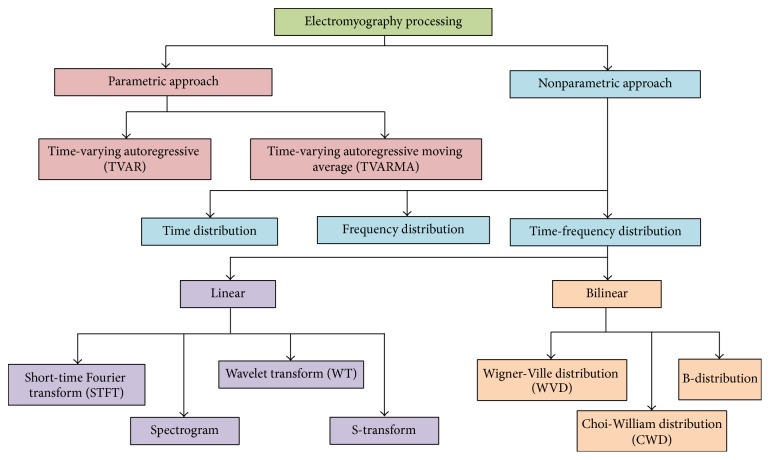
Overall view of the electromyography signal processing methods that are reviewed in this manuscript.

Past researchers have developed several techniques to process the nonstationary signal specifically the EMG signal and these include parametric and nonparametric approaches [40, 41]. The differences between these two approaches are mainly due to the parameters involved. Since most of the signals encounters are unknown signals without known parameters, the development of nonparametric approach, which involves a variety of transforms, is actively researched compared to the parametric approach that requires the modeling of the nonstationary process.  Figure 1 provides an overview of the EMG signal processing methods that are reviewed in the manuscript.

### 6.1. Parametric Approach

In the field of EMG processing, one type of parametric approach that has been explored is based on the time-varying linear predictive models. This includes time-varying autoregressive (TVAR) model where its parameters vary with time. The time-varying spectrum in TVAR is estimated from the time-varying model parameters and the nonstationary signal's instantaneous frequency (IF) can be extracted [42]. IF represents the spectral peak location of the EMG signal as it varies with time [43]. Even though the poles and zeros of the estimated model can be estimated directly from the EMG signal, it is not guaranteed that the time-varying poles remain inside the unit circle on the *z*-plane, thus making TVAR model temporarily unstable [44]. The constraints can be easily imposed by factorizing the denominator of TVAR in direct form and formulating it in cascaded form [45]. Computation of the mean frequency (MNF) is taken as the time average of the IF estimation based on the cascaded model in each interval [46].

Research conducted by Al Zaman et al. has compared TVAR with short-time Fourier transform (STFT) and concludes that even though both TVAR and STFT have produced similar decreasing muscle fatigue patterns from the MNF, TVAR has achieved a better performance with the slope and interception of the linear regression line is closer [47].

Another parametric approach that has been used is the time-varying autoregressive moving average (TVARMA). Studies have been made to compare the performance of TVAR and TVARMA in analyzing EMG signals, and it is concluded that AR models are more favorable than ARMA because of the following reasons: (a) estimation of AR models is moderately basic since they are represented by an arrangement of direct mathematical statements; (b) AR models are predominant in displaying the low recurrence sEMG segment; and (c) fluctuation of the AR model estimation on shorter fragments is lower than for ARMA models [40].

### 6.2. Nonparametric Approach

Another EMG processing method is the nonparametric approaches, which include STFT, spectrogram, and wavelet transform. These approaches appear to be more favorable to researchers since the fatigues indices can be obtained directly from the EMG signal without knowing its parameters. The analysis of the EMG signal based on the nonparametric approaches can be further divided into three types of distribution techniques: time domain, frequency domain, and time-frequency domain [48–50].

#### 6.2.1. Time Distribution (TD)

Extraction of time domain fatigue indices is very simple and easy and involves low computational complexity compared to the other two techniques since it does not involve any transformation [51]. It can be directly obtained from the time representation of the raw EMG signal just by doing some simple mathematical statistics. The idea of fatigue is approximately connected with the amount and rate of change of some variables that reflect muscle alterations amid sustained contractions [52].

Listed in Table 2 are several fatigue indices in the time domain that have been explored by previous researchers. Even though the analysis based on time domain is already established and are widely used since the last decade, however it is considered less accurate since their calculations are based on EMG signal amplitude values, where there are much interference acquired through the recording, especially for features that are extracted from energy property [53]. Another major disadvantage of this time domain features that affects the accuracy comes from the nonstationary property of the EMG signal where the statistical properties changes over time, but time domain features assume the data as stationary signal [54].

Nowadays, researches involving time distribution only focus on finding new fatigue indices with higher robustness, while most of other researchers nowadays opt for time-frequency analysis, which is considered to be more accurate.

#### 6.2.2. Frequency Distribution (FD)

In many studies of fatigue muscle, frequency domain features are usually used to extract information from a signal. To date, there are more than 20 fatigue indices in the frequency domain, where two of the widely used parameters are the mean frequency (MNF) and median frequency (MDF) [75]. Other fatigue indices are listed in Table 3. Several techniques have been used to extract information from EMG signals such as fast Fourier transform (FFT), power spectral density (PSD), and parametric methods (i.e., AR model).

The EMG signal should undergo Fourier transform in order to represent the signal in frequency domain. The definition of Fourier transform in the form of power spectrum is shown in

(1) $$\begin{array}{c} S_{x}\left(\;f\;\right)=\frac{1}{T}{\left|\;\int_{-\infty }^{\infty }x\left(\;t\;\right)\cdot e^{-2\pi ft}dt\;\right|}^{2}, \end{array}$$

where *x*(*t*) is the signal in time domain.

However, the fluctuations of the EMG frequency component due to the adjustments in the muscle force, length and contraction speed throughout time, have caused challenges in the usage of FFT and other traditional processing methods. The fundamental confinement of a FFT is that it cannot give simultaneous time and frequency localization [83]. Therefore, investigation of the EMG signal in dynamic contraction (i.e., repetitive lifting) utilizing these methods may not be successful since it requires the signal to be stationary.

#### 6.2.3. Time-Frequency Distribution (TFD)

Time-frequency representation (TFR) of a signal maps a one-dimensional signal of time into a two-dimensional of time and frequency. In analyzing, modifying, and synthesizing nonstationary signals, TFR are widely used since both representation of time and frequency are taken into account, thus leading to higher accuracy [84].

In general, TFDs consist of linear TFDs and bilinear (quadratic) TFDs. The most basic form of TFD technique is the short-time Fourier transform (STFT). STFT is one of the linear TFDs that have been used to analyze EMG signals along with spectrogram, wavelet transform, S-transform, and so forth. Examples of the bilinear TFDs include the Wigner-Ville distribution (WVD) and Choi-William distribution (CWD).

*Linear TFD*

*Short-Time Fourier Transform (STFT)*. To overcome the disadvantages of time distribution and frequency distribution and satisfy the stationary condition, it is common to separate long haul signals into blocks of narrow fragments [85]. This ought to be sufficiently narrow to be viewed as stationary and take the FT of every segment. Every FT gives the spectral information of a different time-slice of the signal, giving simultaneous time and frequency estimation.

This was proposed by Gabor who had developed the STFT, the extended version of FT [86].

(2) $$\begin{array}{c} {\text{STFT}}_{x}\left(\;t,w\;\right)=\overset{\infty }{\underset{-\infty }{\int }}x\left(\;\tau \;\right)w\left(\;\tau -t\;\right)e^{-2\pi f\tau }d\tau , \end{array}$$

where *x*(*τ*) is the EMG signal, *w*(*τ* − *t*) is the observation window, and *t* is the variable that slides the window over the signal, *x*(*τ*).

The choice of the window function in STFT is critical in order to get accurate results. The shape of the window can be either rectangular, Gaussian, or elliptic, depending on the shape of the signal. For sampled STFT using a Gaussian window, it is often called Gabor transform. Even though the window should be narrow enough to ensure that the portion of the signal that falls within the window is stationary, it must not be too narrow since it will lead to bad localization in the frequency domain. For infinitely long window, *w*(*t*) = 1 will eventually cause STFT to turn into FT, providing excellent frequency localization, but no time localization. In contrast to infinitely short window, *w*(*t*) = *δ* will result in the time signal (with a phase factor), which will provide excellent time localization but no frequency localization.

Research by MacIsaac et al. uses MNF as fatigue index and has drawn three important conclusions. First, the nonstationarities in EMG do not seem to affect the MNF values obtained from Fourier transform. Secondly, the STFT method to measure MNF has successfully midpoints out the impacts of nonstationarities in dynamic compressions. Third, the STFT is capable of identifying a descending pattern with fatigue in dynamic contraction, as long as the range of motion remains consistent across sequential time interval [87].

Since the STFT is basic in idea and execution and is equipped for distinguishing a pattern in muscle fatigue, it is an undeniable choice for applications that require simple analysis with acceptable results.

*Spectrogram*. The squared magnitude of the STFT is called spectrogram. It can be expressed as

(3) $$\begin{array}{c} S_{x}\left(\;t,f\;\right)={\left|\;\int_{-\infty }^{\infty }x\left(\;\tau \;\right)w\left(\;\tau -t\;\right)e^{-2\pi f\tau }\;\right|}^{2}d\tau , \end{array}$$

where *x*(*τ*) is the EMG signal, *w*(*τ* − *t*) is the observation window, and *t* is the variable that slides the window over the signal, *x*(*τ*).

Spectrogram can be used to obtain the power distribution and energy distribution of the signal along the frequency direction at a given time. In EMG processing, the instantaneous energy is useful to separate muscle activation from the baseline, which is called the segmentation process. Segmentation is important in reducing the high computational complexity of TFDs. The muscle activation segmentation process by using spectrogram has been proposed by Shair et al. and resulted in a mean absolute percentage error (MAPE) of just 1.404% [88].

For both STFT and spectrogram, there is a compromise between the time-based and frequency-based perspective of a signal. Both time and frequency are represented in limited precision, where precision is controlled by the span of the window, and the size of the window chosen will be the same for all frequencies.

In 2010, Janković and Popović have presented in their paper the use of spectrogram in the analysis of muscle fatigue. They highlighted that even though the traditional MDF technique provides good sensitivity for fatigue indication, however, there is a significant slope error particularly for low activity of coinitiated muscles. They proposed a hybrid of alternative methods (spectrogram and scalogram) that can successfully provide complete information but at the same time produce less error prediction for low-level coinitiated muscles [89]. As a recommendation for improvement, they also proposed further investigation on the robustness of this method in dynamic exercise contractions.

Later in 2016, Zawawi et al. came out with a detailed analysis of EMG signals by using spectrogram for manual lifting application. By taking the average instantaneous RMS voltage (*V*_rms_(*t*)) as fatigue index, she concludes that, as the lifting height is increased, the average *V*_rms_(*t*) is also increased. However, as the average *V*_rms_(*t*) decreased, the number of lifting repetitions is increased [90].

*Wavelet Transform (WT)*. A wavelet is a waveform of effectively limited duration that has an average value of zero. Some of its properties are short-time localized waves with zero integral value, the possibility of time shifting, and flexibility. Wavelet analysis produces a time-scale view of the signal. The result of a continuous wavelet transform (CWT) is wavelet coefficients. Multiplying each coefficient with the appropriately scaled and shifted wavelet yields the constituent wavelet of the original signal. Equation (4) shows the formula for CWT.

(4) $$\begin{array}{c} {\text{CWT}}_{x}=\overset{\infty }{\underset{-\infty }{\int }}x\left(\;\tau \;\right)\frac{1}{\sqrt{\left|a\right|}}\Psi \left(\;\frac{\tau -t}{a}\;\right)d\tau , \end{array}$$

where *t* is the translation, *a* is the scale parameter, and Ψ is the mother wavelet.

Yochum et al. have proposed a new fatigue index based on the continuous wavelet transform (CWT), named *I*_CWT_, and have compared it to other fatigue indices from literature in terms of their sensitivity to noise. The results show that this new fatigue index quantifies the EMG signal elongation during a contraction and thus makes it a suitable fatigue index. *I*_CWT_ is less subjected to noise and less truncation dependent compared to other fatigue indices based on the frequency like MNF and MDF [91].

A comparison has been made by Dantas et al. between STFT and CWT in evaluating muscle fatigue in isometric and dynamic contractions. The after effects of this study exhibit that CWT and STFT analysis give comparable fatigue estimates (slope of MDF) in isometric and dynamic contractions. However, the aftereffects of CWT for both contractions indicate less variability (higher accuracy) in EMG signal analysis contrasted with STFT [92].

*S-Transform*. Another TFD technique exists is the S-transform. This technique has been widely used in diverse areas of telecommunication, power quality, geophysics, and biomedicine, due to its obvious advantages in processing nonstationary signals [93]. In the area of EEG and ECG, despite the use of S-transform in the time-frequency analysis, this technique is also a good alternative for denoising and removal of artifacts that exist in ECG and EEG signals [94]. Even though it has been explored in the areas of EEG and ECG, there are no available researches conducted in utilizing this technique for EMG signal.

S-transform was introduced in 1996 by Stockwell, Mansinha, and Lowe. It is a hybrid of two advanced signal processing techniques which are STFT and WT [95]. Due to this, it inherits the good qualities from both techniques. It provides good resolution in both time and frequency and allows users to assess any frequency component in the time-frequency domain without the need of using any digital filter. Even though S-transform uses a variable window length to maintain a good time-frequency resolutions for all frequencies, it still retains the phase information using a Fourier kernel.

Expression for S-transform is shown in

(5) $$\begin{array}{c} {\text{ST}}_{x}\left(\;t,f\;\right)=\overset{\infty }{\underset{-\infty }{\int }}x\left(\;\tau \;\right)\frac{\left|f\right|}{\sqrt{2\pi }}e^{-{\left(\tau -t\right)}^{2}\tau^{2}/2}e^{-j2\pi f\tau }d\tau , \end{array}$$

where *τ* and *f* denote the time of the spectral localization and Fourier frequency, respectively.

The development of the S-transform is led by the urge to overcome the low resolution of STFT and the absence of the phase information in CWT. Since the features had existed in the S-transform, it is sometimes viewed as a variable sliding window STFT or a phase-corrected CWT [96].

Although S-transform has better time-frequency resolution than STFT, the resolution is still far from perfect and needs improvement. To date, there are several improved S-transform introduced by researchers such as the modified S-transform [96] and discrete orthonormal S-transform [94].

*Bilinear TFD*

*Wigner-Ville Distribution (WVD)*. Wigner first introduced WVD in the area of quantum mechanics in the year 1932, and later in 1948, Ville developed and applied the same transformation to signal processing and spectral analysis [97].

Compared to STFT and WT, WVD does not contain a windowing function and thus frees WVD from the smearing effect due to the windowing function. As a result, it provides best possible temporal and frequency resolution in the time-frequency plane. It possesses several interesting properties, such as energy conservation, real-valued, marginal properties, translation covariance, dilation covariance, instantaneous frequency, and group delay.

However, because of its quadratic nature, when dealing with signals with several frequency components, WVD suffers from the so-called cross components (inference terms), which represent significant defects in this method.

These interference terms are troublesome since they may overlap with autoterms (signal terms) and in this manner, it is hard to visually interpret the WVD image. It creates the impression that these terms must be available or the good properties of WVD (localization, group delay, instantaneous frequency, marginal properties, etc.) cannot be fulfilled. There is also a trade-off between the amount of interferences and the number of good properties. These are known issues with the WVD spectrum, and there are several ways to compensate these cross terms that has been discussed by previous researchers [98–100]. Equation (6) shows the formula for WVD.

(6) $$\begin{array}{c} W_{x}\left(\;t,f\;\right)=\overset{\infty }{\underset{-\infty }{\int }}x\left(\;t+\frac{\tau }{2}\;\right)x^{*}\left(\;\tau -\frac{\tau }{2}\;\right)e^{-2j\pi f\tau }d\tau , \end{array}$$

where *x*(*t* + *τ*/2)*x*^*∗*^(*τ* − *τ*/2) is the instantaneous autocorrelation function and *∗* shows conjugate operation.

In the occasion that time smoothing window *g*(*t*) and a frequency smoothing window *h*(*t*) are connected to WVD, WVD will then transform into the smoothed-pseudo-Wigner-Ville distribution (SPWVD), as composed in

(7) SPWVDxt,f=∬ht−τgf−εWxτ,εdτ dε,

where *W* is the WVD.

A research by Subasi and Kiymik in 2010 has compared the performance between STFT, SPWVD, and CWT in terms of accuracy, specificity, and sensitivity. The results suggested that all three methods provide almost similar performance and are found to be satisfactory, despite the fact that there are little differences between the results [99].

*Choi-William Distribution (CWD)*. CWD is a member of Cohen's class distribution function and was proposed in the year 1989 by [101]. It is able to avoid one of the main problems of WVD, which is the presence of interference in regions where one would expect zero power values. Adoption of the exponential kernel in the distribution helps to suppress the effect of cross term [102]. CWD is given by

(8) CWDxt,f=∫−∞∞∫−∞∞Axη,τΦη,τej2πηt−τfdη dτ,

where

(9) $$\begin{array}{c} A_{x}\left(\;\eta ,\tau \;\right)=\overset{\infty }{\underset{-\infty }{\int }}x\left(\;t+\frac{\tau }{2}\;\right)x^{*}\left(\;t-\frac{\tau }{2}\;\right)e^{-j2\pi t\eta }dt \end{array}$$

and the kernel function is given by

(10) $$\begin{array}{c} \Phi \left(\;\eta ,\tau \;\right)=e^{-{\alpha \left(\eta \tau \right)}^{2}}. \end{array}$$

By studying the influence of muscle contraction towards the frequency content of EMG signal using WVD and CWD, Alemu et al. observed that cross terms existing in WVD are greatly reduced by CWD, thus resulting in easier interpretation with no loss of definition due to cross terms [103]. The reduction of the cross terms is due to the smoothing parameters introduced in CWD. However, this still depends on the smoothing parameter value selected. As observed from the result itself, when the smoothing parameter decreased, the reduction of the cross terms is better. This, however, affects the time and frequency resolutions by reducing it and leads to more signal loss. As the smoothing parameter approaches *∞*, it resembles WVD more [103].

*B-Distribution*. Karthick et al. introduced B-distribution in 2015 to attenuate the cross terms exist in WVD and CWD. This is realized by introducing a smoothing function referred to as quadratic time-frequency distribution (QTFD) [104]. Expression of the QTFD is given as follows [105]:

(11) pn,k=2∑m<M/2Gn,m∗zn+mz∗n−me−j2πkm/M,

where *G*[*n*, *m*] is the smoothing kernel and operator *∗* represents convolution.

Kernel of the B-distribution is given by

(12) $$\begin{array}{c} G\left[\;n,m\;\right]={\left(\;\frac{\left|2m\right|}{\cosh^{2}n}\;\right)}^{\beta }*\left(\;\sin cm\;\right), \end{array}$$

where *β* is a smoothing parameter.

Three fatigue indices, namely, instantaneous median frequency (IMDF), instantaneous mean frequency (IMNF), and instantaneous spectral entropy (ISPEn), were used alongside the B-distribution and it is found that all three features are distinct in both fatigue and nonfatigue conditions. The study concludes that B-distribution may be useful in EMG analysis under various clinical and normal conditions.

*Modified B-Distribution*. A year later, the same author came out with a study using modified B-distribution to monitor the progression of muscle fatigue. Since the performance of time-frequency distribution is assessed based on its ability to reduce cross term and provide closely spaced frequency components representation, this modified version successfully removes cross term interference, while maintaining high time-frequency resolution [106]. It is demonstrated that modified B-distribution based time-frequency distribution performs better in suppressing cross terms with good time and frequency resolution for multicomponent signals compared with other above-mentioned techniques [107]. Recently, this technique has been widely used to analyze nonstationarities related to EEG signals, heart rate signals, and accelerometer data based on fetal movements [108, 109].

## 7. Discussion and Conclusion

The use of EMG processing to assess muscle fatigue during manual lifting was reviewed and several fatigue indices were introduced. Research in the area of EMG signal currently evolves around the sensitivity, variability, and repeatability of the fatigue indices, as well as the best processing techniques with high efficiency and less computational complexity. Despite the use of traditional methods such as time distribution and frequency distribution, time-frequency distribution is found to be more superior in terms of monitoring since it enables the user to observe the progress of the signal in time and frequency. This is very important as at the point when muscle fatigue sets in, frequency compression can happen. Unlike the conventional frequency distribution technique, time-frequency analysis demonstrates the time when any changes in frequencies occur.

In recent years, researchers had identified that the bilinear TFD could perform better than the linear TFD such as STFT, spectrogram, and WT due to the fact that it does not suffer from the smearing effects cause by windowing function. Nonetheless, on account of its quadratic nature, bilinear TFD suffers the cross term effects. With the existence of high resolution Cohen class TFD such as the modified B-distribution introduced in 2016, the cross term effects can be removed and at the same time maintain the high time-frequency resolution.

Other than the processing techniques listed in this paper, there are still several techniques such as Hilbert-Huang transform, which have been used to analyze the EMG signals. There are also already well established techniques in other research areas and are proven to be effective and have yet to be implemented in analyzing EMG signals such as the S-transform. This technique may be suitable for nonstationary signals like EMG itself, due to its good nature, including linearity, lossless reversibility, multiple resolution, good time-frequency resolution, and simple algorithm. In conclusion, it can be seen that there are many gaps to be filled in this area in either finding new and better fatigue indices or constantly developing new and improved processing techniques.

## Figures and Tables

**Table 1:** Common muscles investigated during manual lifting.

Reference	Muscle areas
Kamarudin et al. [7]	Biceps brachii and triceps brachii
Voge and Dingwell [8]	Biceps, middeltoid, midtrapezius, and postdeltoid
Zawawi et al. [9]	Biceps brachii
Al-Ashaik et al. [10]	Biceps brachii, erector spinae, anterior deltoid, and trapezius
Granata and Marras [11]	Erector spinae, external abdominal obliques, internal abdominal obliques, rectus abdominis, and latissimus dorsi
Roy et al. [12]	Longissimus thoracis, iliocostalis lumborum, and multifidus
Seroussi and Pope [13]	Erector spinae and external oblique
Graham et al. [14]	Lumbar erector spinae, thoracic erector spinae, and rectus abdominis
Gagnon et al. [15]	Thoracic erector spinae, lumbar erector spinae, internal oblique, external oblique, rectus abdominis, and latissimus dorsi
Dolan and Adams [16]	Erector spinae
Bonato et al. [17]	Iliocostalis lumborum, longissimus thoracis, and multifidus
Potvin [18]	Thoracic erector spinae and lumbar erector spinae
Kingma and Van Dieën [19]	External oblique, internal oblique, rectus abdominis, iliocostalis lumborum, longissimus thoracis, and pars thoracis
Shin and Kim [20]	Erector spinae, rectus abdominis, latissimus dorsi, internal oblique, and external oblique
Cholewicki et al. [21]	Lumbar erector spinae, thoracic erector spinae, latissimus dorsi, internal oblique, external oblique, and rectus abdominis

**Table 2:** Fatigue indices in time domain.

Reference	Fatigue indices
Malinzak et al. [55]	Integrated EMG (IEMG)
Arabadzhiev et al. [56]	Root mean square (RMS)
Suetta et al. [57]	Mean absolute value (MAV)
Oskoei and Hu [58]	Modified mean absolute value type 1 (MAV1)
Villarejo et al. [59]	Modified mean absolute value type 2 (MAV2)
Phinyomark et al. [60]	Simple integral square (SSI)
Zardoshti-Kermani et al. [61]	Variance of EMG (VAR)
Tkach et al. [62]	v-order (V)
Zardoshti-Kermani et al. [61]	Log detector (LOG)
Kiguchi et al. [63]	Waveform length (WL)
Al Omari et al. [64]	Average amplitude change (AAC)
Siddiqi et al. [65]	Difference absolute standard deviation value (DASDV)
Kilbom et al. [66]	Zero crossing (ZC)
AlOmari and Liu [67]	Myopulse percentage rate (MYOP)
Phinyomark et al. [68]	Willison amplitude (WAMP)
Rogers et al. [69]	Slope sign change (SSC)
Buchenrieder [70]	Mean absolute value slope (MAVSLP)
Venugopal et al. [71]	Multiple hamming windows (MHW)
Du and Vuskovic [51]	Multiple trapezoidal windows (MTW)
Phinyomark et al. [72]	Histogram of EMG (HIST)
Al-Quraishi et al. [73]	Autoregressive coefficient (AR)
Chang et al. [74]	Cepstral coefficient (CC)

**Table 3:** Fatigue indices in frequency domain.

Reference	Fatigue indices
Merletti and Lo Conte [76]	Mean frequency (MNF)
De Luca [77]	Median frequency (MDF)
Khanam and Ahmad [78]	Peak frequency (PKF)
Khanam and Ahmad [78]	Mean power (MNP)
Khanam and Ahmad [78]	Total power (TTP)
Altaf et al. [79]	The 1st, 2nd, and 3rd spectral moments (SM1, SM2, and SM3)
Phinyomark et al. [80]	Power spectrum ratio (PSR)
González-Izal et al. [81]	Instantaneous frequency variance (*F*_var_)
Georgakis et al. [75]	Averaged instantaneous frequency (AIF)
Dimitrov et al. [82]	Dimitrov's spectral fatigue index (FI_nsm5_)
